# Isolation of *Bacillus* sp. A5.3 Strain with Keratinolytic Activity

**DOI:** 10.3390/biology11020244

**Published:** 2022-02-04

**Authors:** Saniya Aktayeva, Kairat Baltin, Assel Kiribayeva, Zhiger Akishev, Dmitriy Silayev, Yerlan Ramankulov, Bekbolat Khassenov

**Affiliations:** 1 Laboratory of Genetics and Biochemistry of Microorganisms, National Center for Biotechnology, Nur-Sultan 010000, Kazakhstan; aktayevasa@gmail.com (S.A.); Baltin@biocenter.kz (K.B.); kiribayeva@biocenter.kz (A.K.); akishev@biocenter.kz (Z.A.); silayev@biocenter.kz (D.S.); ramanculov@biocenter.kz (Y.R.); 2 Department of General Biology and Genomics, L.N. Gumilyov Eurasian National University, Nur-Sultan 010000, Kazakhstan

**Keywords:** feathers, protease, keratinase, *Bacillus*, proteomics

## Abstract

**Simple Summary:**

In this study, we described keratinolytic properties of a strain of *Bacillus* (sp. A5.3) isolated from sites of feather waste accumulation. The proteolytic enzymes secreted by *Bacillus* sp. A5.3 are serine proteases, are alkaline enzymes, have a wide substrate specificity, and have high thermal stability. *Bacillus* sp. A5.3 effectively hydrolyzes feathers and can be used in feather-processing technologies and as a source of alkaline and thermostable proteases and keratinases.

**Abstract:**

Environmental safety and economic factors necessitate a search for new ways of processing poultry farm feathers, which are 90% β-keratin and can be used as a cheap source of amino acids and peptones. In this study, feather-decomposing bacteria were isolated from a site of accumulation of rotten feathers and identified as *Bacillus*. Among them, the *Bacillus* sp. A5.3 isolate showed the best keratinolytic properties. Scanning electron microscopy indicated that *Bacillus* sp. A5.3 cells closely adhere to the feather surface while degrading the feather. It was found that *Bacillus* sp. A5.3 secretes thermostable alkaline proteolytic and keratinolytic enzymes. Zymographic analysis of the enzymatic extract toward bovine serum albumin, casein, gelatin, and β-keratin revealed the presence of proteases and keratinases with molecular weights 20–250 kDa. The proteolytic and keratinolytic enzymes predominantly belong to the serine protease family. Proteome analysis of the secreted proteins by nano-HPLC coupled with Q-TOF mass spectrometry identified 154 proteins, 13 of which are proteases and peptidases. Thus, strain *Bacillus* sp. A5.3 holds great promise for use in feather-processing technologies and as a source of proteases and keratinases.

## 1. Introduction

Feather biomass consists of 90% of protein [1], which is a valuable biological product, and feather hydrolysates can be a source of peptones [2,3]. The feather is rich in essential amino acids leucine, valine, arginine, isoleucine, phenylalanine, and threonine, with smaller proportions of lysine, methionine, histidine, and tryptophan [4,5]. Sulfur-containing cysteine and methionine, together with threonine, tyrosine, and phenylalanine, are important for the biosynthesis of hair and feather keratin [6]. Unfortunately, feather proteins consist of insoluble protein extensively cross-linked by disulfide bonds, e.g., β-keratin [7].

Due to a sharp reduction in biological resources, the use of feather hydrolysates as a source of amino acids and peptones is relevant for biotechnological industries [8]. On the other hand, feather keratin is insoluble in water and has low digestibility by enzymes of the pepsin family due to disulfide bonds, hydrogen bonds, and hydrophobic interactions between its amino acid residues [9]. For enzymatic hydrolysis of keratin, keratinases capable of cleaving disulfide bonds are employed. Keratinases (EC 3.4.99.11) are serine- or metalloproteases [10], and a large number of microorganisms have been reported to produce keratinases [11]. Bacteria show the greatest promise for the keratin-processing technology. Therefore, just as the preparation of keratin-degrading microorganisms, the research on the genes of proteins with keratinolytic activity is relevant and promising. The resultant knowledge will allow to obtain recombinant-enzyme preparations for use in the technologies for processing of low-value feather material into a valuable protein product [12].

Familiarity with the problem of feather waste recycling on poultry farms prompted us to find solutions to this problem. The technology used for processing feathers into fodder by mechanical grinding and heat treatment, in our opinion, is not an optimal solution because β-keratin, due to its stability, is hardly degraded and is not digested in the alimentary tract of birds. Moreover, feather powder clogs the intestines of the birds and makes it difficult to digest the feed [13]. Therefore, we aimed to find a bacterial producer of keratinolytic enzymes for subsequent use in technologies for organic processing of feather waste.

In this work, we isolated bacteria capable of growing on a feather medium from sites of accumulation of feather waste on a poultry farm. A *Bacillus* strain (sp. A5.3) with suitable proteolytic and keratinolytic properties was identified and chosen as a potential producer of enzymes. Temperature and pH characteristics, substrate specificity, and composition of the enzymatic extract were studied. Our data indicate that *Bacillus* sp. A5.3, as a producer of effective keratinases, is a promising candidate for use in technologies for enzymatic processing of feather waste.

## 2. Materials and Methods

### 2.1. Chemicals

Ovalbumin was acquired from MP Biomedicals (USA), whereas bovine serum albumin (BSA) and casein sodium salt and keratin azur from Sigma (St. Louis, MO, USA). Phenylmethylsulphonyl fluoride (PMSF), pepstatin A, ethylenediaminetetraacetic acid (EDTA), media ingredients, and chemical reagents were purchased from Sigma and AppliChem (Darmstadt, Germany).

### 2.2. Culture Media

The basic medium that was utilized for the isolation and fermentative cultivation of feather-degrading microorganisms (Feather broth) was composed of the following constituents (g/L): NaH_2_PO_4_ (0.3), Na_2_HPO_4_ (0.35), and feather (7.5), pH 7.0. A feather agar medium and milk agar were used for screening the microorganisms on plates. For feather agar, feather powder was prepared according to the following protocol: chicken feathers were washed with a detergent and chloroform and dried at 120 °C for 8 h. The dried feathers were cut and chopped in a ball mill. A feather agar medium contained the following constituents (g/L): KH2PO4 (0.7), Na2HPO4 (1.4), feather powder (5), and agar (20) [14].

Nutrient broth (0.5% [*w*/*v*] of peptone, 0.5% [*w*/*v*] of NaCl, 0.15% [*w*/*v*] of yeast extract, and 0.15% [*w*/*v*] of beef extract, pH 7.4) and nutrient agar were employed to cultivate isolates. The Luria-Bertani (LB) medium (1% [*w*/*v*] of trypton plus, 0.5% [*w*/*v*] of yeast extract, and 0.5% [*w*/*v*] of NaCl, pH 7.2) was employed for inoculum preparation and isolate maintenance.

### 2.3. Isolation and Identification of Microorganisms

Rotten feathers and soil samples were collected at a local poultry plant. One gram of a sample with 9 mL of 0.9% (*w*/*v*) NaCl was shaken for 30 min, and 100 µL of the suspension was seeded in feather agar plates, followed by cultivation at 37 °C for 48 h. Well-grown stand-alone colonies were isolated and tested for a proteolytic activity on a milk agar plate. Plates were grown for 8 h at 37 °C and followed by 16 h incubation at RT (23–24 °C) for clearing zones appear. Activity-positive colonies were again seeded in feather agar plates. A single colony on a plate was chosen randomly and subjected to identification and further analyses.

Morphological characteristics of each isolate were compared with data from Bergey’s Manual of Systematic Bacteriology. Genomic DNA from the cells was isolated with the Wizard Genomic DNA Purification Kit (Promega, Madison, WI, USA). A fragment of the 16S rRNA gene was amplified by PCR with a universal primer pair (27F [5′-AGAGTTTGATCCTGGCTCAG-3′] and 1492R [5′-TACGGTTACCTTGTTACGACTT-3′]) and sequenced. The sequences were compared with GenBank data using the Basic Local Alignment Search Tool (BLAST; http://blast.ncbi.nlm.nih.gov/Blast.cgi accessed on: 20 December 2018 and 13 January 2022). Identification by matrix-assisted laser desorption ionization (MALDI) time-of-flight (TOF) mass spectrometry (MS) was performed by means of Biotyper Microflex LT (Bruker Daltonics, Bremen, Germany).

### 2.4. Preparation of an Extracellular Enzymatic Extract

Cells of a strain were inoculated into 5 mL of the LB medium and were cultured overnight at 37 °C and 200 rpm in a shaking incubator. The overnight culture was inoculated into 150 mL of Feather broth and cultured for 48 h. The supernatant was clarified by centrifugation (10,000× *g*, 4 °C, 10 min) and passed through a membrane with 0.22 μm pore size to remove microparticles and bacteria. The enzymatic extract was stored on ice and used for keratinolytic and proteolytic assays.

### 2.5. Azokeratin Preparation

Azokeratin was prepared according to a published protocol [15]. Chicken feathers were washed with a detergent and an ethanol–chloroform mixture (1:1 ratio) and were dried at room temperature. The feathers were cut and powdered in the ball mill. One gram of the feather powder was added to 20 mL of MilliQ water with 10% (*w*/*v*) of NaHCO3, and the mixture was stirred. A solution was prepared separately: 174 mg of sulfanilic acid was dissolved in 5 mL of 0.2N NaOH, and 69 mg of NaNO2 and 0.4 mL of 5 M HCl were added, stirred for 2 min, and the solution was neutralized by the addition of 0.4 mL of 5N NaOH. This solution was added to the feather suspension, and the mixture was stirred for 10 min. The pellet was collected and suspended in water (that was prewarmed to 50 °C), stirred for 2 h, and the suspension was filtered through 0.45 μm. The insoluble pellet was washed with 50 mM potassium phosphate buffer (pH 7.5) and lyophilized at −90 °C in vacuum.

### 2.6. An Assay of Enzymatic Activity

Keratinolytic activity was measured according to Lin et al. [16] using azokeratin as the substrate. In brief, 5 mg of azokeratin was dissolved in 0.8 mL of 50 mM sodium phosphate buffer (pH 7.5). Next, 0.2 mL of the enzymatic extract was added to the azokeratin solution. The mixture was incubated at 50 °C for 1 h (standard assay conditions). The reaction was stopped with 0.2 mL of 10% (*w*/*v*) trichloroacetic acid (TCA). The mixture was centrifuged for 10 min at 10,000× *g*. The absorbance of the supernatant was measured at 450 nm using an appropriate blank (10% TCA was added to the mixture before incubation). An absorbance increase of 0.01 units at 450 nm was defined as 1 U of keratinolytic activity. Proteolytic activity was measured as described previously [17] by means of casein as a substrate. A standard curve was constructed by means of a 0–100 mg/L solution of tyrosine. One unit of proteolytic activity was defined as the amount of the enzymatic extract needed to liberate 1 μg of tyrosine per minute under the experimental conditions.

### 2.7. Effects of Temperature on Enzyme Activity and Stability

The keratinolytic and proteolytic activities were measured in the temperature range of 30–80 °C (with 10 °C intervals) in 50 mM Tris–HCl buffer (pH 7.5) and 50 mM glycine–NaOH buffer (pH 11.0), respectively. Maximum enzymatic activity was set to 100% activity. To investigate thermostability, an enzyme solution was preincubated for 3 h at 50 °C, 60 °C, and 70 °C in an optimal buffer, and then the residual activity was quantified. The keratinolytic activity was measured at optimal temperature and pH.

### 2.8. Effects of pH on Enzyme Activity and Stability

The enzymatic activity was measured in the pH range 3.0 to 12.0 (with one or half pH unit intervals) for 1 h on azokeratin and for 15 min on casein at optimal temperature. Maximum enzymatic activity was set to 100% activity. To determine the effect of pH on enzyme stability, the enzymatic extract was preincubated in buffers having different pH levels (6–11) for 5 h. The keratinase activity was measured at optimal temperature and pH. The following buffer systems were utilized: citrate buffer (pH 3.0–6.0), sodium phosphate buffer (pH 6.0–7.5), Tris-HCl (pH 7.5–9.0), glycine-NaOH (pH 9.0–11.0), and potassium chloride buffer (pH 12.0).

### 2.9. The Impact of Metal Ions, Protease Inhibitors, and Chemical Reagents on the Keratinase Activity

Effects of metal ions (K^+^, Na^+^, Li^+^, Cs^+^, Ni^2+^, Mg^2+^, Ca^2+^, Cd^2+^, Zn^2+^, Mn^2+^, Cu^2+^, Fe^3+^, or Al^3+^), protease inhibitors (PMSF, EDTA, or pepstatin A), chemical surfactants and detergents (1% *v*/*v*; Tween 20, Triton X-100, or SDS), and reducing and oxidizing agents (β-mercaptoethanol, dithiothreitol [DTT], or H_2_O_2_) on the keratinase activity were tested next. Keratinase activity was measured by preincubation of the enzymatic extract with a reagent for 30 min at 37 °C, and then enzymatic activity toward azokeratin was assayed. The keratinase activity in the absence of metal ions was regarded as 100%.

### 2.10. Zymograms with Different Substrates

The ability of the enzymatic extract to degrade various substrates was evaluated by SDS-PAGE in a 4–20% gel with (*w*/*v*) a copolymerized casein sodium salt (0.1%), BSA (0.1%), gelatin (1%), or β-keratin (0.7%) incorporated as a substrate. The enzymatic extract was mixed with SDS-PAGE sample buffer (125 mM Tris-HCl pH 6.8, 4% of SDS, 0.002% of bromophenol, and 20% of glycerol) in a sample:buffer ratio of 4:6. Next, PMSF (5 mM), EDTA (5 mM), or pepstatin A (0.035 mM) was added to the sample. The samples were not boiled before loading on the gel, and β-mercaptoethanol was not added into the loaded samples before they were subjected to electrophoresis. The gels were washed twice for 10 min in 2.5% Triton X-100 and then incubated in 0.5 M Tris-HCl pH 8.5 at 50 °C for 20 h. The gels were stained with 0.08% Coomassie Brilliant Blue G-250 in 20% ethanol with 1.6% phosphoric acid and 8% ammonium sulfate for 4 h and destained in MilliQ water. Protein markers (New England Biolabs, cat. # P7719S) were used for molecular mass determination.

### 2.11. Hydrolysis of Different Substrates

BSA, ovalbumin, casein sodium salt, β-keratin, and keratin azure (α-keratin) were separately tested as substrates for hydrolysis by the enzymatic extract of *Bacillus* sp. A5.3. Substrates were dissolved in 1 mL of 50 mM Tris-HCl pH 8.5 to attain 0.25 mg/mL concentration for BSA, 0.5 mg/mL for ovalbumin, 1 mg/mL for casein, and 1 mg/mL for keratin and then were treated with the enzymatic extract at 60 °C. A substrate without the enzymatic extract served as a control. After hydrolysis, SDS-PAGE in a 4–20% gel was performed as described by Laemmli [18]. The gels with BSA, ovalbumin, and casein were stained for 2 h with 10% acetic acid in 50% ethanol containing 2% of Coomassie Brilliant Blue R-250. The gel with β-keratin was stained with silver according to Bassam et al. [19]. The protein markers (New England Biolabs cat. # P7719S and Sigma cat. # M3546) were utilized for molecular mass determination. The keratinolytic activity toward keratin azure was assayed in accordance with Vermelho et al. [20].

### 2.12. Feather Degradation

A strain inoculum was grown in the LB medium for 16 h at 37 °C and 200 rpm. Next, 1 mL of the inoculum was added into a glass test tube with a chicken feather in 50 mM sodium phosphate buffer (pH 7.5). Degradation was conducted on a shaker (250 rpm) at 37 °C for 216 h. Feather degradation was determined by means of the dry weight of the remaining feather in the medium during the incubation as follows. The culture was passed through pre-weighed filter paper, and the retained residue was washed twice with distiller water and dried at 60 °C until constant weight. The result was expressed as a percentage of the initial feather weight (100%).

### 2.13. Scanning Electron Microscopy (SEM) Analysis of Feather Degradation

Samples of the feather from the inoculated and control (without the microorganism) culture were removed after 2 and 6 days of incubation at 37 °C with shaking (300 rpm) and were examined by SEM regarding feather degradation. Feather samples were placed on stubs and gold-sputtered (10 nm). Images were captured by means of an Auriga Crossbeam 540 (Carl Zeiss, Jena, Germany) scanning electron microscope operating at 3 kV.

### 2.14. Nano-High-Performance Liquid Chromatography Coupled with Quadrupole TOF (NanoHPLC-Q-TOF) MS and Mascot Analysis

The enzymatic extract was concentrated on a Pierce^TM^ Protein Concentrator (10K MWCO: 10 kDa molecular weight cutoff) by 50-fold. Electrophoretic separation of proteins was performed by SDS-PAGE in a 12% polyacrylamide gel. The proteins were extracted from the gel samples and trypsinized. Peptides were separated on an Acclaim Pep-Map RSLC column (Thermo Scientific, Waltham, MA, USA) with an acetonitrile gradient. An unmodified CaptiveSpray ion source was employed to interface the HPLC system with a Maxis Impact II instrument (Bruker). The mass range of the MS scan was set to m/z 150–2200 in positive ion polarity mode. The Mascot software was used to perform searches against the SwissProt 2016_08 database (552,884 sequences; 197,760,918 residues).

### 2.15. Statistical Analysis

All experiments were conducted in triplicate. For quantitative assays, mean values and standard deviations (SDs) were calculated using the GraphPad Prism Version 8.0.1 software in this work. Enzymatic activity is presented as the mean, and other values are shown as the mean ± SD (*n* = 3).

## 3. Results

### 3.1. Isolation and Identification of the Keratinolytic Strain

In the present work, four bacterial strains were isolated from rotten-feather and soil samples collected on a local poultry farm: A5.3, A5.5, A7.1, and A11.2. The strains were found to be capable of growing on a minimal salt medium with feather as the only source of carbon and nitrogen.

**Figure 1:**
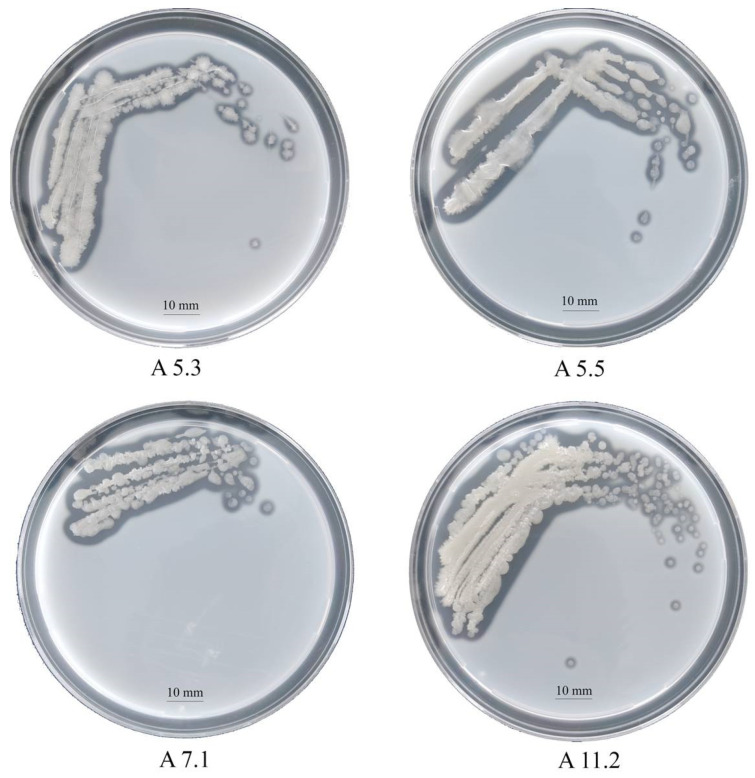
Colonies of *Bacillus* isolates A5.3, A5.5, A7.1, and A11.2 on a skim milk agar plate.

On nutrient agar after 24 h, the strains formed beige colonies, 1–2 mm in diameter with wavy edges. The cells turned out to be Gram-positive, oval, mobile, and forming spores. By sequencing and comparing the fragment of 16S rRNA gene nucleotide sequence with GenBank data, we identified the strains as bacteria of the genus *Bacillus*. The proteomic profiling of ribosomal proteins carried out on the Biotyper Microflex LT instrument confirmed that the studied strains belong to the genus *Bacillus* (Supplementary Materials Table S1). The milk agar test revealed that all the strains have a proteolytic activity (Figure 1). Clear zones around the colonies formed due to the hydrolysis of milk casein and were clearly visible on the plates. The following results on the clear zone size were obtained: A5.3, 2.9 mm; A5.5, 1.9 mm; A7.1, 1.5 mm; and A11.2, 1.8 mm.

**Figure 2:**
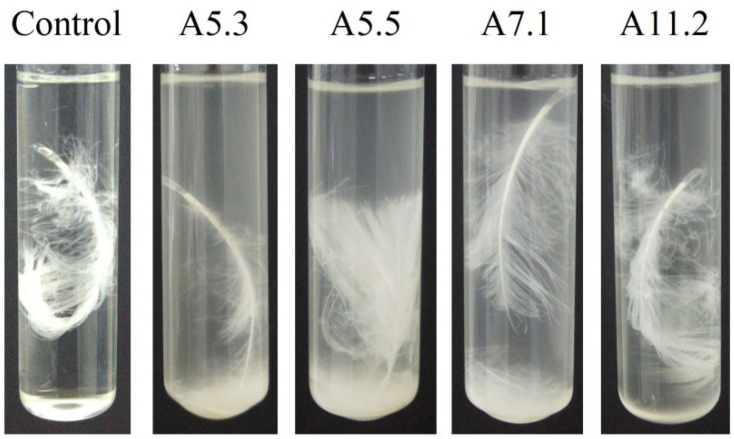
Feather degradation by *Bacillus* isolates A5.3, A5.5, A7.1, and A11.2.

Cultivation of the isolates on the feather medium for 96 h showed that *Bacillus* sp. A5.3 possesses the highest activity (Figure 2).

In further experiments, isolate *Bacillus* sp. A5.3 was chosen as the strain producing keratinolytic and proteolytic enzymes. The enzymatic extract for further experiments here was obtained by collecting and filtering a supernatant after the cultivation of *Bacillus* sp. A5.3 on the minimal feather medium.

### 3.2. Effects of Temperature and pH on Enzymatic Activity

**Figure 3:**
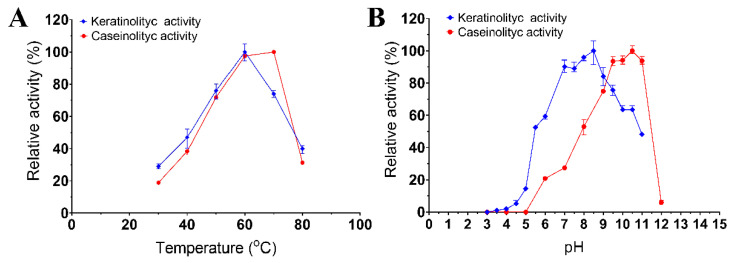
Effects of temperature (**A**) and pH (**B**) on keratinolytic and caseinolytic activities. Values shown in (**A**,**B**) (mean ± standard deviation [SD]) are representative data obtained in triplicate assays of the enzymatic extract from *Bacillus* sp. A5.3.

The enzymatic extract of *Bacillus* sp. A5.3 was found to have a temperature optimum at 60 °C for keratinolytic activity and 70 °C for caseinolytic activity (Figure 3A). The enzymatic extract of strain A5.3 has a pH optimum of 8.5 and 10.5 for keratinolytic and caseinolytic activity, respectively (Figure 3B). The enzymatic extract showed >75% of keratinolytic activity in the pH range 7.0–9.5 and 75% of caseinolytic activity in the pH range 9.0–11.0. The highest activity of the enzymatic extract from *Bacillus* sp. A5.3 under optimal conditions proved to be 109.3 ± 4.0 and 158.8 ± 2.5 U/mL for keratinolytic and caseinolytic activity, respectively.

### 3.3. The Impact of Temperature and pH on Enzyme Stability

**Figure 4:**
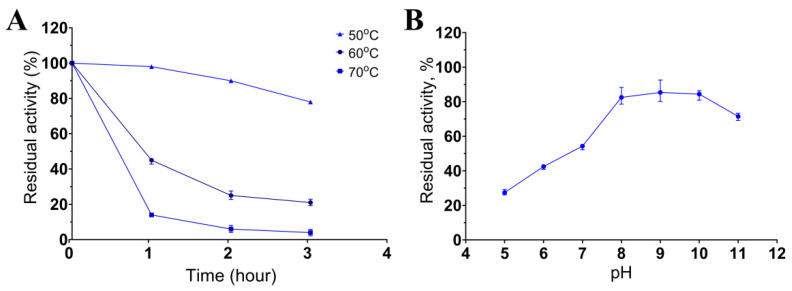
The influence of temperature (**A**) and pH (**B**) on keratinase stability. Values shown in (**A**,**B**) (mean ± standard deviation [SD]) are representative data obtained in triplicate assays of the enzymatic extract from *Bacillus* sp. A5.3.

Keratinase stability was determined after incubation of the enzymatic extract at 50, 60, or 70 °C and pH 8.5 for 3 h. It retained 80% of activity after 3 h at 50 °C and 45% and 21% of activity after incubation at 60 °C for 1 and 3 h, respectively (Figure 4A). The enzymatic extract was more sensitive to the temperature of 70 °C: incubation at this temperature already after 1 h reduced the activity to 14%, and after 3 h of incubation, the activity was ≤4% of the initial activity. The enzymatic activity was determined in the pH range of 5 to 11 after 5 h incubation (Figure 4B). The extract retained over 80% of the initial activity between pH 8.0 and 11.0. The results about the dependence of the keratinase activity on temperature and pH indicated that the keratinases of *Bacillus* sp. A5.3 are thermostable and alkaline enzymes.

### 3.4. Effects of Metal Ions, Detergents, Reducing Agents, and Inhibitors

The keratinases in the enzymatic extract from *Bacillus* sp. A5.3 were inhibited by metal ions having a single, double, or triple charge (Table 1): by threefold by Mn^2+^; twofold by K^+^, Li^+^, Mg^2+^, and Cd^2+^; by 35–40% by Cs^+^, Ni^2+^, Zn^2+^, Fe^3+^, and Al^3+^; and slightly by Na^+^ and Cu^2+^. Keratinases from *Bacillus* sp. A5.3 were inhibited by Tween 20, ¦ Â-mercaptoethanol, DTT, H_2_O_2_, and EDTA and proved tolerant to Triton X-100. SDS inhibited the keratinolytic activity by 10%. Pepstatin A had no significant effect on the keratinolytic activity of the enzyme solution, and PMSF diminished the keratinase activity of the enzymatic extract by 70% (Table 1).

### 3.5. Substrate Specificity

**Figure 5:**
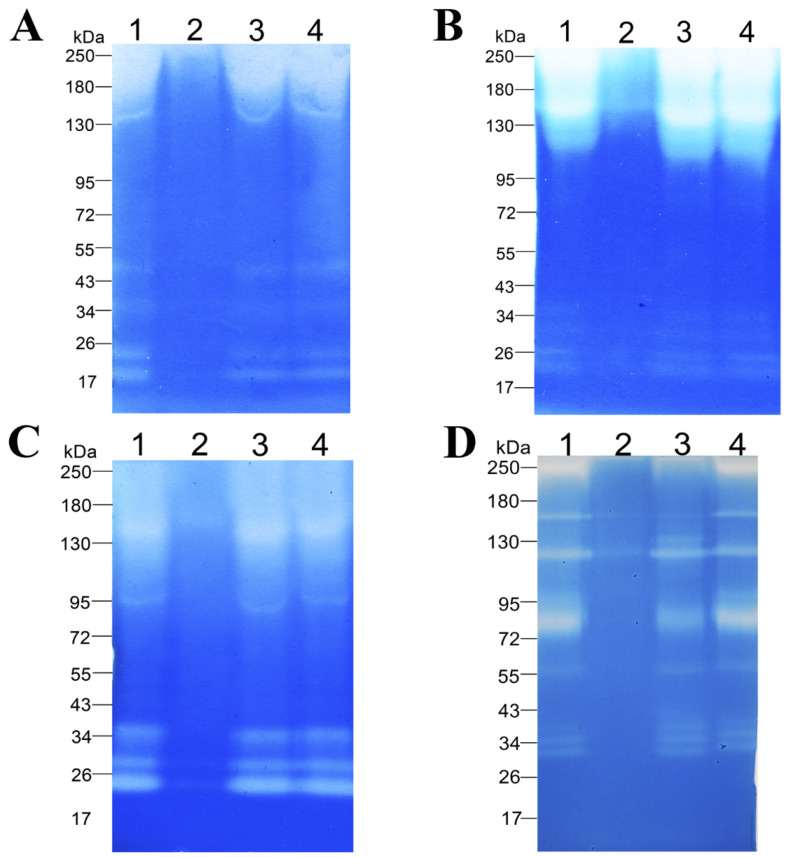
Zymograms with copolymerized casein (**A**), BSA (**B**), gelatin (**C**), or β-keratin (**D**). Lane 1, Enzymatic extract; lane 2, enzymatic extract with PMSF; lane 3, enzymatic extract with EDTA; and lane 4, enzymatic extract with Pepstatin A.

The ability of the enzymatic extract to degrade proteinaceous substrates from various classes was demonstrated on casein (Figure 5A, Supplementary Materials Figure S1A), BSA (Figure 5B, Supplementary Materials Figure S1B), gelatin (Figure 5C, Supplementary Materials Figure S1C), and feather keratin (Figure 5D, Supplementary Materials Figure S1D) copolymerized with sodium dodecyl sulfate polyacrylamide gels. The figure shows that enzymes of different molecular weights in the range of 20–250 kDa are involved in the hydrolysis of these substrates. The zymogram with casein showed six bands migrating approximately at 20, 22, 34, 50, 150, and 250 kDa (Figure 5A, Supplementary Materials Figure S1A). The zymogram with BSA also contains six bands migrating at the levels of approximately 20, 26, 30, 36, 150, and 250 kDa (Figure 5B, Supplementary Materials Figure S1B). The zymogram with gelatin contains five bands migrating at approximately 22, 28, 34, 95, and 150 kDa (Figure 5C, Supplementary Materials Figure S1C). The zymogram with β-keratin features nine bands migrating at approximately 30, 34, 38, 55, 75, 95, 115, 150, and 250 kDa. In the zymographic analysis, inhibitory effects of protease inhibitors (PMSF [lane 2], EDTA [lane 3], and pepstatin A [lane 4]) were studied too. Readers can see that PMSF strongly inhibits the proteolytic and keratinolytic activity of the enzymatic extract (Figure 5, Supplementary Materials Figure S1). The enzymatic extract of *Bacillus* sp. A5.3 did not show any activity toward keratin azure.

**Figure 6:**
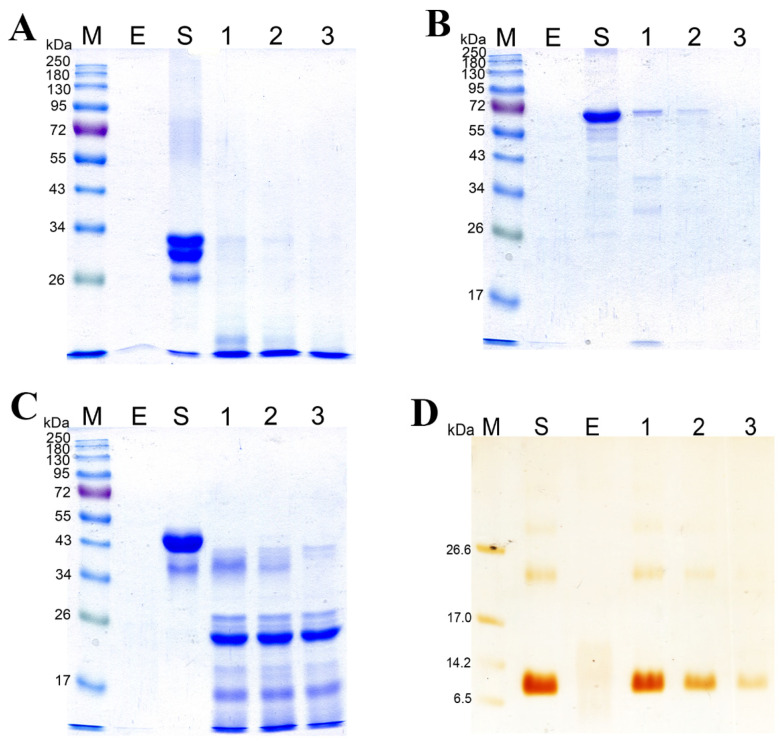
Degradation of the casein sodium salt (**A**), BSA (**B**), ovalbumin (**C**), and β-keratin (**D**) by the enzymatic extract from *Bacillus* sp. A5.3 depending on incubation time. M, protein molecular weight markers. E, enzymatic extract without a substrate; S, substrate. In (**A**): lane 1, 15 s; lane 2, 30 s; and lane 3, 60 s. In (**B**): lane 1, 5 min; lane 2, 15 min; and lane 3, 60 min. In (**C**): lane 1, 1 min; lane 2, 5 min; and lane 3, 15 min. In (**D**): lane 1, 30 min; lane 2, 60 min; and lane 3, 120 min.

Figure 6 shows the data on SDS-PAGE of the products of hydrolysis of substrates: casein sodium salt, BSA, gelatin, and β-keratin. It was demonstrated experimentally that the rates of hydrolysis of various substrates by the enzymatic extract are different. The casein sodium salt was found to be hydrolyzed faster than other substrates (Figure 6A, Supplementary Materials Figure S2A); it began degrading after 15 s of treatment with the enzymatic extract and was completely hydrolyzed in 1 min. BSA and ovalbumin were completely hydrolyzed by the enzymes in 60 and 15 min, respectively (Figure 6B,C, Supplementary Materials Figure S2B,C). β-Keratin was found to be the most resistant to the action of the enzymatic extract (Figure 6D, Supplementary Materials Figure S2D). Even after 2 h of digestion, β-keratin was hydrolyzed incompletely.

### 3.6. Feather Degradation and SEM Analysis

**Figure 7:**
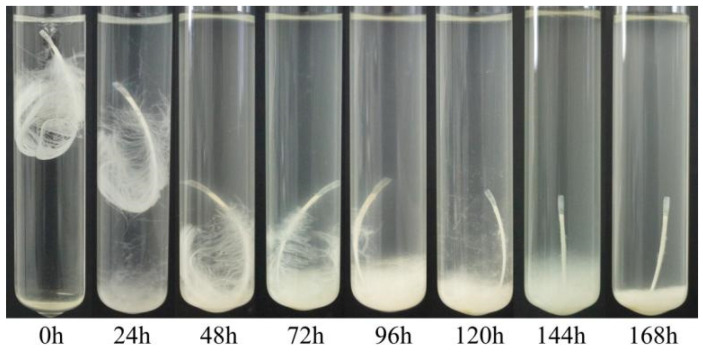
Feather degradation by *Bacillus* sp. A5.3 depending on incubation time.

Our different strains showed dissimilar activity in terms of feather degradation. Treatment of a chicken feather with a culture of *Bacillus* sp. A5.3 for 7 days showed that the strain effectively degrades up to 70% of the feather and completely degrades it within 9 days (Figure 7). After 2 days, degradation of barbules was observed, and degradation of barbs started on the third day. The degradation of barbs continued throughout the incubation period and resulted in complete exposure of the shaft. The culture became milky.

**Figure 8:**
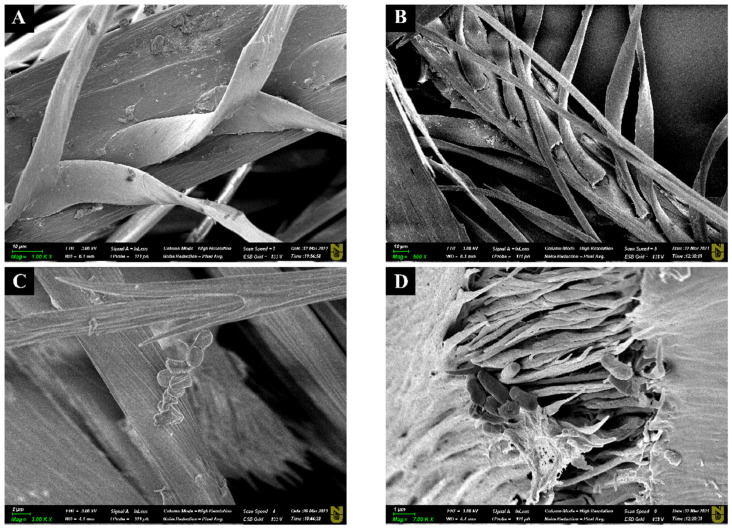
Scanning electron microscopy (SEM) images of chicken feather, where (**A**) is the intact feather, (**B**,**D**) show the feather after 48 h of incubation, and (**C**) is the feather after 144 h of incubation. Scale bars in (**A**–**C**), 10 μm; and in (**D**), 1 μm.

The degradation of chicken feather by *Bacillus* sp. A5.3 after 2 and 4 days of cultivation was demonstrated by SEM (Figure 8). An untreated sample had intact barb structure (Figure 8A).

Damaged barbs were visible after 48 h (Figure 8B). After 144 h, the structure of the barbs was even more damaged (Figure 8C). When the resolution was increased, the bacteria adhering to the surface of the feathers were clearly visible (Figure 8D).

### 3.7. NanoHPLC-Q-TOF Identification of Enzymes

To identify these secretory proteins, chromate-MS analysis of the *Bacillus* sp. A5.3 culture supernatant was performed. Supernatant proteins were trypsinized, and the peptide mixture was separated by nanoHPLC. The peptides were electrospray-ionized, parental and daughter ions were obtained, and their spectra were determined on the Q-TOF mass spectrometer. Bioinformatics analysis of the spectra on the Mascot platform against the SwissProt database indicated that 154 proteins were present in the culture supernatant of strain *Bacillus* sp. A5.3; 36 of these proteins were similar to those of *B. subtilis* strain 168. In this study, 13 proteins of interest (proteases and peptidases) were found (Supplementary Materials Table S2).

## 4. Discussion

Bacilli have a large number of proteolytic enzymes, which allow them to occupy various ecological niches [21]. Bacilli are usually found in soils, various composts, and waste sites and are predominantly mesophilic microorganisms, but the enzymes secreted by them have high thermostability [22,23]. The good productivity properties of bacteria from the genus *Bacillus* have allowed this taxon to assume a dominant position in the microbial synthesis industry [24]. Among keratin-degrading microorganisms, Bacilli are known producers of keratinases [25], and several keratinolytic bacteria have been isolated from soil and poultry wastes [26].

We isolated *Bacillus* sp. A5.3 from rotten feathers collected on a poultry farm and studied its keratinolytic and proteolytic properties. Bacteria producing keratinases are known to exist [27,28,29]; however, the focus of research has been on the isolation, purification, and characterization of properties of individual enzymes.

Good growth of bacteria does not always indicate high production capacity of the strains. In a study by Ghosh et al. [30], only 29 (76%) of 38 isolates showed proteolytic potential, and in a study by Torres de Oliveira et al. [31], only 6 (40%) out of 15 isolates were capable of casein degradation. Screening for feather degradation is more vivid, but the cultivation of isolates on feather agar as proposed previously [31,32] did not allow us to clearly see the hydrolysis zones. Cultivation of the isolates in a liquid salt medium with a feather and screening for the degree of feather degradation turned out to be more informative. Cultivation of the isolates on the feather medium for 96 h showed that *Bacillus* sp. A5.3 possesses the highest activity. Cultivation of four strains (A5.3, A5.5, A7.1, and A11.2) on feather medium showed that the *Bacillus* sp. A5.3 has the highest keratinolytic activity among all.

On the other hand, in the research on new keratinolytic microorganisms, it is preferable to characterize an enzyme solution because the synthesis of various proteolytic enzymes will allow for complete hydrolysis of keratin. In a practical application, it is easier and cheaper to obtain a total enzymatic extract. A few investigators have studied enzymatic extracts of bacteria regarding keratin degradation [33,34]. The biochemical characteristics of the enzymatic extract of *Bacillus* sp. A5.3 indicate that the keratinases produced by the strain are alkaline and thermostable enzymes with a maximum keratinolytic activity at pH 8.5 and 60 °C. A comparative analysis revealed that most of the bacterial keratinases are also alkaline enzymes and have an optimum of 40–50 °C (Table 2). The same optimum as in *Bacillus* sp. A5.3 (60 °C) has been documented for *Nocardiopsis* sp. TOA-1, *B. licheniformis* RPk, *B. subtilis* MA21, *B. zhangzhouensis* BK111, *Serratia* sp. HPC 1383, *Brevibacillus parabrevis* CGMCC 10798, and *B. halodurans* PPKS-2.

An assay of the proteolytic activity of the enzymatic extract of *Bacillus* sp. A5.3 toward casein showed that the proteases secreted by the strain have an even higher alkalinity and temperature optimum: pH 10.5 and 70 °C, respectively. In comparison, keratinases from *B. licheniformis* RPk and *B. subtilis* NRC3 have a caseinolytic optimum of 65 and 40 °C, respectively [28,36]. In addition, keratinases from *Bacillus* sp. A5.3 are highly thermally stable, retaining 80% of activity after 3 h at 50 °C or 45% of activity after 1 h at 60 °C. Keratinases from *Bacillus* sp. A5.3 are also highly pH stable, retaining more than 80% of activity in the pH range 8.0–10.0.

For keratinases from *Bacillus* sp. A5.3, all the studied metal ions had negative effects on the activity. Cu^2+^ also had inhibitory effects on the keratinolytic activity from *Bacillus* sp. A5.3 but not so strongly as described by Nam et al. [43]. A similar inhibitory effect on keratinases from *Bacillus* sp. A5.3 was exerted by Mn^2+^, as is the case for keratinase from *B. subtilis* KD-N2 [27], and conversely, not the case for keratinase from *Fervidobacterium islandicum* AW-1 [43], on which Mn^2+^ exerts a stimulatory effect. Some *Bacillus* keratinases can be partially inhibited by EDTA owing to the importance of cations as stabilizing agents in these keratinases [10]. A negative impact of EDTA on keratinase activity has also been documented for keratinases from *B. halodurans* PPKS-2 [42], *Bacillus* sp. MKR5 [44], and *Chryseobacterium* sp. kr6 [45], and on the contrary, a positive effect of EDTA is known for keratinase from *B. subtilis* KD-N2 [27]. The observed negative impact of EDTA is possibly related to our finding that metalloproteases are present among the keratinases in the enzymatic extract from *Bacillus* sp. A5.3, and their inhibition reduces the total keratinase activity. The nonionic detergent Triton X-100 either does not affect the keratinase activity of these enzymes, as described by Prakash et al. [42], or increases it, as per Younes et al. [44] and Riffel et al. [45]. Most keratinases from *Bacillus* spp. that have been described are affiliated with the serine peptidase class. Therefore, PMSF is a likely inhibitor of these enzymes. Keratinolytic activity of the enzymatic extract from *Bacillus* sp. A5.3 was strongly inhibited by PMSF as reported for other keratinases from *Bacillus* representatives [26,39,42,46,47,48]. The other proteases are likely to be metalloproteases because in the presence of EDTA, the activity of the enzymatic extract diminished too. Pepstatin A did not have an inhibitory influence on the enzymatic extract from *Bacillus* sp. A5.3, indicating the absence of aspartyl proteases among the enzymes in the extract.

Substrate specificity is one of the major criteria used for designating any protease as a “keratinase.” Keratinases are known to efficiently cleave materials that are high in keratin content, e.g., feathers, nails, wool, and soluble substrates such as casein, azocasein, and BSA [49]. To distinguish keratinases from other conventional proteases, it has been suggested that their K:C (keratinolytic/caseinolytic) activity ratio should be employed as an indicator of an enzyme’s efficiency as a keratinase [50]. Enzymatic extract of *Bacillus* sp. A5.3 manifested 109.3 ± 4.0 and 158.8 ± 2.5 U/mL keratinolytic and caseinolytic activities, respectively, meaning that the K:C ratio is 0.69; an enzyme can be considered a potential keratinase when this ratio > 0.5 [49]. For comparison, K:C ratios of nonkeratinolytic enzymes trypsin, papain, and proteinase-K are 0.08, 0.02, and 0.28, respectively [50]. Our zymographic characterization of the *Bacillus* sp. A5.3 enzymatic extract uncovered broad substrate specify. Zymographic analysis of the enzymatic extract toward bovine serum albumin, casein, gelatin, and β-keratin revealed the presence of proteases and keratinases with molecular weights 20–250 kDa. A similar zymogram with many bands has been reported for *B. subtilis* SCL and *B. subtilis* 1271 [33,39]. It should be noted that according to the assay of the activity toward keratin azure, the enzymatic extract does not contain keratinases that hydrolyze α-keratin. This can be explained by the source of the *Bacillus* sp. A5.3 strain: feathers, not wool or hair.

The addition of protease inhibitors—PMSF, EDTA, or pepstatin A—revealed that proteases of this enzymatic extract are strongly inhibited only by PMSF. The keratinases of the extract are strongly inhibited by PMSF and partly by EDTA. This means that these keratinases are affiliated with the serine protease family and metalloprotease family.

Among the substrates, casein is the most sensitive to the action of the enzymatic extract of *Bacillus* sp. A5.3: 1 mg of the soluble casein salt was completely hydrolyzed in 1 min; 0.5 mg of ovalbumin was hydrolyzed within 15 min; 0.25 mg of BSA was hydrolyzed twice as long; 1 mg of β-keratin was hydrolyzed much more slowly due to its high resistance to proteolytic enzymes. This finding is explained by the large number of disulfide bonds cross-linking polypeptide chains, by hydrophobic interactions, and by hydrogen bonds in the structure of β-keratin.

Treatment of a chicken feather with a culture of *Bacillus* sp. A5.3 for 9 days completely degraded it. For complete degradation of feathers, it is preferable to use live bacteria, not purified keratinases. Extracellular keratinolytic activity of *B. licheniformis* RG1 without live bacteria cells fails to degrade a feather [7]. In that study, structural analysis showed that these bacteria closely adhere to barbules and produce a keratinase that diffuses laterally while degrading the rachis and barbules [7]. Our experiments also suggest that the enzymatic extract without live bacteria did not degrade feathers completely. SEM data indicate that *Bacillus* sp. A5.3 cells closely adhered to the feather surface while degrading the feather. Similar results have been obtained elsewhere [7,33]. The bacterial adhesion plays an important role in the feather degradation process.

In the mass spectrometric analysis of the secretory proteome of *Bacillus* sp. A5.3, we identified 13 proteases and peptidases. It seems interesting to study in vitro these 13 proteases and peptidases with putative keratinase activity. Genes of three proteases—*clpY*, *clpX*, and *ytjP*—have already been successfully amplified, cloned, sequenced by us. Their nucleotide sequences were deposited in GenBank (accession numbers: MW276067, MW276068 and MW276069). We are planning on investigating these and 10 other enzymes from the enzymatic extract of *Bacillus* sp. A5.3 in future projects.

## 5. Conclusions

In this study, *Bacillus* sp. A5.3 was isolated from rotten feathers and has the highest keratinolytic activity among isolates. The keratinases and proteases in the *Bacillus* sp. A5.3 enzymatic extract are thermostable alkaline enzymes and have activities 109.3 ± 4.0 and 158.8 ± 2.5 U/mL, respectively. Keratinolytic enzymes of *Bacillus* sp. A5.3 belong to serine protease and metalloprotease families. Cultivation of *Bacillus* sp. A5.3 on the chicken feather revealed that the strain completely degrades the feather within 9 days. SEM data indicate that *Bacillus* sp. A5.3 cells closely adhere to the feather surface while degrading the feather. This work may offer effective bacterial keratinases for enzymatic hydrolysis of feathers in terms of processing of poultry farm waste and production of protein feed additives.

## Figures and Tables

**Table 1:** Effects of various chemicals on keratinolytic activity of the *Bacillus* sp. A5.3 extract.

Chemicals	Concentration	Residual Activity, %
None		100 ± 3.3
K^+^	5 mM	57.2 ± 6.0
Na^+^	5 mM	82.3 ± 3.6
Li^+^	5 mM	46.3 ± 4.8
Cs^+^	5 mM	62.7 ± 7.8
Ni^2+^	5 mM	60.8 ± 2.1
Mg^2+^	5 mM	49.6 ± 2.7
Ca^2+^	5 mM	71.1 ± 6.4
Cd^2+^	5 mM	49.0 ± 2.9
Zn^2+^	5 mM	65.4 ± 6.6
Mn^2+^	5 mM	35.3 ± 3.3
Cu^2+^	5 mM	81.1 ± 3.8
Fe^3+^	5 mM	68.1 ± 1.0
Al^3+^	5 mM	75.2 ± 1.5
Tween 20	1%, *v*/*v*	57.7 ± 6.7
Triton X-100	1%, *v*/*v*	98.1 ± 7.7
SDS	1%, *w*/*v*	90.2 ± 5.6
Β-mercaptoethanol	5 mM	57.7 ± 6.7
Dithiothreitol	5 mM	52.5 ± 2.1
H_2_O_2_	1 M	68.9 ± 4.6
EDTA	5 mM	79.1 ± 5.3
PMSF	5 mM	30.9 ± 3.2
Pepstatin A	35 µM	100.0 ± 2.9

**Table 2:** Comparison of biochemical properties of various bacterial keratinases with properties of the enzymatic extract from *Bacillus* sp. A5.3.

Strain	Temperature Optimum, °C	pH Optimum	Reference
*Bacillus* sp. A5.3	60	8.5	Present study
*B. pumilus* AR57	45	9.0	[35]
*B. subtilis* NRC3	50	7.5	[36]
*B. licheniformis* PWD-1	50	7.5	[16]
*B. subtilis* KD-N2	55	8.5	[27]
*Nocardiopsis* sp. TOA-1	60	12.5	[37]
*B. licheniformis* RPk	60	9.0	[28]
*B. subtilis* MA21	60	9.0	[38]
*B. zhangzhouensis* BK111	60	9.5	[29]
*B. subtilis* 1271	40–50	10	[39]
*B. licheniformis* 1269	40–50	10	[39]
*B. cereus* 1268	40–50	10	[39]
*Serratia* sp. HPC 1383	60	10	[40]
*Brevibacillus parabrevis* CGMCC 10798	60	8.0	[41]
*B. halodurans* PPKS-2	60	11	[42]

## Data Availability

All datasets used and/or analyzed during the current study are available from the corresponding author on reasonable request.

## Supplementary Materials

The following are available online at https://www.mdpi.com/article/10.3390/biology11020244/s1, Figure S1: Zymograms with copolymerized casein (**A**), BSA (**B**), gelatin (**C**), or β-keratin (**D**). Lane 1, Enzymatic extract; lane 2, enzymatic extract with PMSF; lane 3, enzymatic extract with EDTA; and lane 4, enzymatic extract with Pepstatin A. Figure S2: Degradation of the casein sodium salt (**A**), BSA (**B**), ovalbumin (**C**), and β-keratin (**D**) by the enzymatic extract from *Bacillus* sp. A5.3 depending on incubation time. M, protein molecular weight markers. E, enzymatic extract without a substrate; S, substrate. In (**A**): lane 1, 15 s; lane 2, 30 s; and lane 3, 60 s. In (**B**): lane 1, 5 min; lane 2, 15 min; and lane 3, 60 min. In (**C**): lane 1, 1 min; lane 2, 5 min; and lane 3, 15 min. In (**D**): lane 1, 30 min; lane 2, 60 min; and lane 3, 120 min. Table S1: Identification results for the isolates according to Biotyper data, Table S2: Proteases and peptidases found in a culture supernatant of *Bacillus* sp. A5.3.
