# Supplementary material for: Isolation of Bacillus sp. A5.3 Strain with Keratinolytic Activity
Source: Biology (Basel). 2022 Feb 4;11(2):244. doi: 10.3390/biology11020244 (PMC8869582; doi:10.3390/biology11020244)
Supplement: Supplementary file 1 [file biology-11-00244-s001.zip › biology-1519818-supplementary.pdf]

Supplementary Materials:

Table S1. Identification results for the isolates according to Biotyper data.

Strain	Organism (Best Match/Second-best Match)	Score
A5.3	*Bacillus vallismortis*	1.981
	Bacillus subtilis	1.952
A5.5	*Bacillus subtilis*	1.819
	Bacillus mojavensis	1.761
A7.1	*Bacillus cereus*	1.879
	Bacillus mycoides	1.812
A11.2	*Bacillus cereus*	2.3
	Bacillus cereus	2.223

Table S2. Proteases and peptidases found in a culture supernatant of *Bacillus* sp. A5.3.

MW (kDa)	Protein	Peptides	Amount	Max. Score	Total Score
85.6	Minor extracellular protease vpr OS=*B. subtilis* (strain 168) GN=vpr PE=1 SV=1	R.VVIPAHQTGK.A	4	48	116
		K.VPTLLIVKEPDYPR.V	12	43	420
		K.GVAPDATLLAYR.V	2	35	66
52.6	ATP-dependent protease ATPase subunit ClpY OS=*B. subtilis* (strain 168) GN=clpY PE=1 SV=1	R.LLVPGKKK.Q	1	26	26
47.6	Uncharacterized protease YrrO OS=*B. subtilis* (strain 168) GN=yrrO PE=3 SV=1	K.MIEMGIDSLK.I + Oxidation (M)	2	29	50
46.3	ATP-dependent Clp protease ATP-binding subunit ClpX OS=*B. subtilis* (strain 168) GN=clpX PE=2 SV=3	R.FGLIPEFIGR.L		27	27
33.8	Major intracellular serine protease OS=B. subtilis (strain 168) GN=isp PE=1 SV=2	K.LTGTSMAAPHVSGALALIK.S + Oxidation (M)	3	33	89
		R.TLPLDIAK.T	2	21	37
64.2	Gamma-glutamyltranspeptidase OS=*B. subtilis* (strain 168) GN=ggt PE=1 SV=1	K.GTAVGVPGTLK.G	1	20	20
		K.DVFLPNGEPLKEGDTLIQK.D	1	24	24
53.7	Probable cytosol aminopeptidase OS=*B. subtilis* (strain 168) GN=pepA PE=3 SV=1	R.LVLADGITYAK.Q	3	56	119
		K.TIEILNTDAEGR.L	5	34	157
51.1	Putative dipeptidase YtjP OS=*B. subtilis* (strain 168) GN=ytjP PE=3 SV=1	K.KADLISIGGGTYAR.S	1	29	29
45.5	Peptidase T OS=*B. subtilis* (strain 168) GN=pepT PE=3 SV=1	R.VAFTPDEEIGRGPHK.F	3	38	82
		-.MKEEIIER.F	1	27	27
		K.AVNVIVEIAK.Q	1	14	14
39.2	Putative aminopeptidase YtoP OS=*B. subtilis* (strain 168)	K.YADDIVQDR.L	3	60	162

	GN=ytoP PE=3 SV=1				
38.7	Putative aminopeptidase YhfE OS=*B. subtilis* (strain 168) GN=yhfE PE=3 SV=1	K.SGHDIVHGLIGPGIDASHAFER.T	5	38	160
		K.IDLIGGFR.Y	1	29	29
38.1	Uncharacterized peptidase YqhT OS=*B. subtilis* (strain 168) GN=yqhT PE=3 SV=1	K.IADDAFR.H	3	31	80
30.2	D-aminopeptidase OS=*B. subtilis* (strain 168) GN=dppA PE=1 SV=3	K.EAEELIPNVTTAAVK.Q	1	27	27

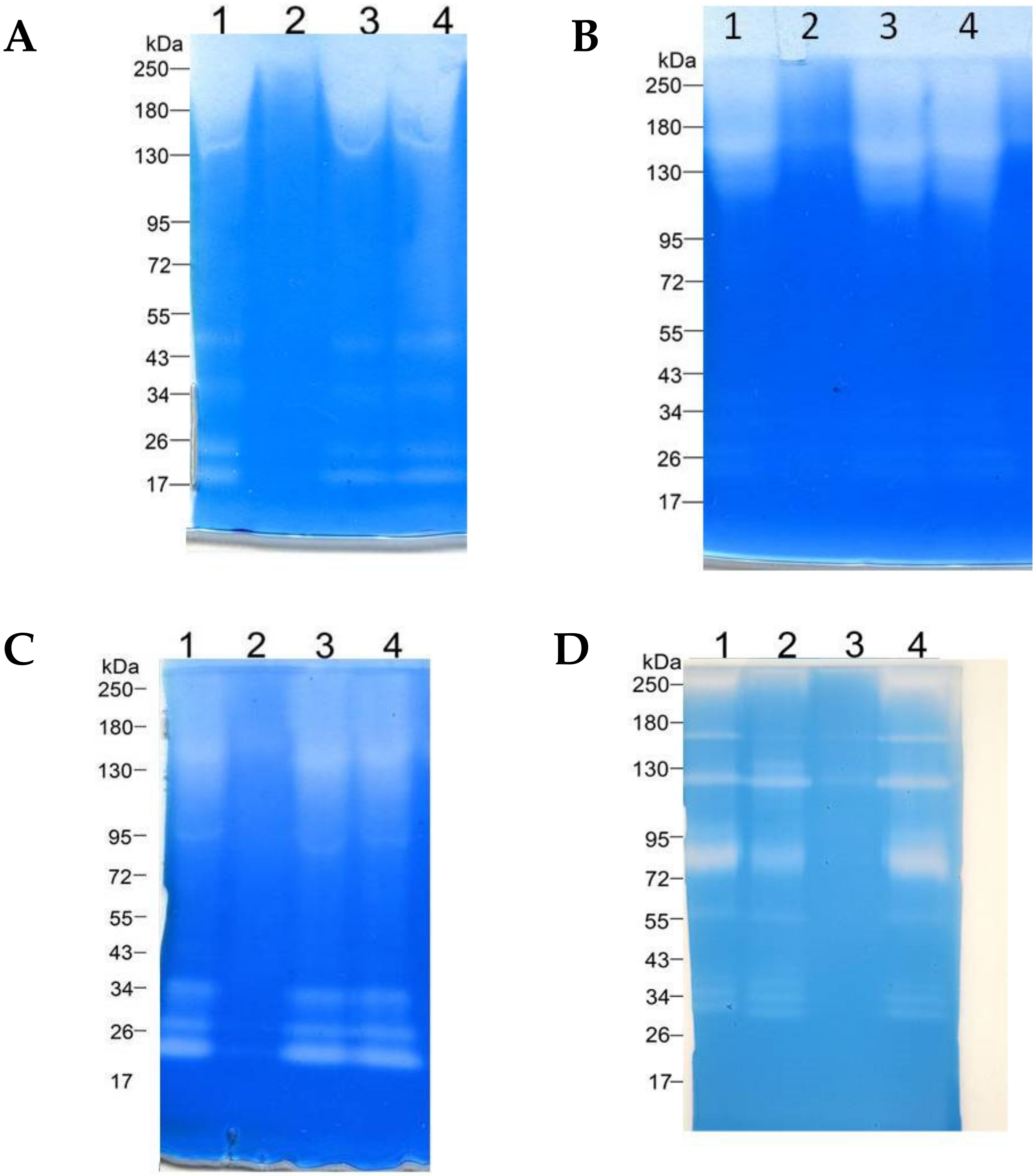

Figure S1. Zymograms with copolymerized casein (**A**), BSA (**B**), gelatin (**C**), or β-keratin (**D**). Lane 1, Enzymatic extract; lane 2, enzymatic extract with PMSF; lane 3, enzymatic extract with EDTA; and lane 4, enzymatic extract with Pepstatin A.

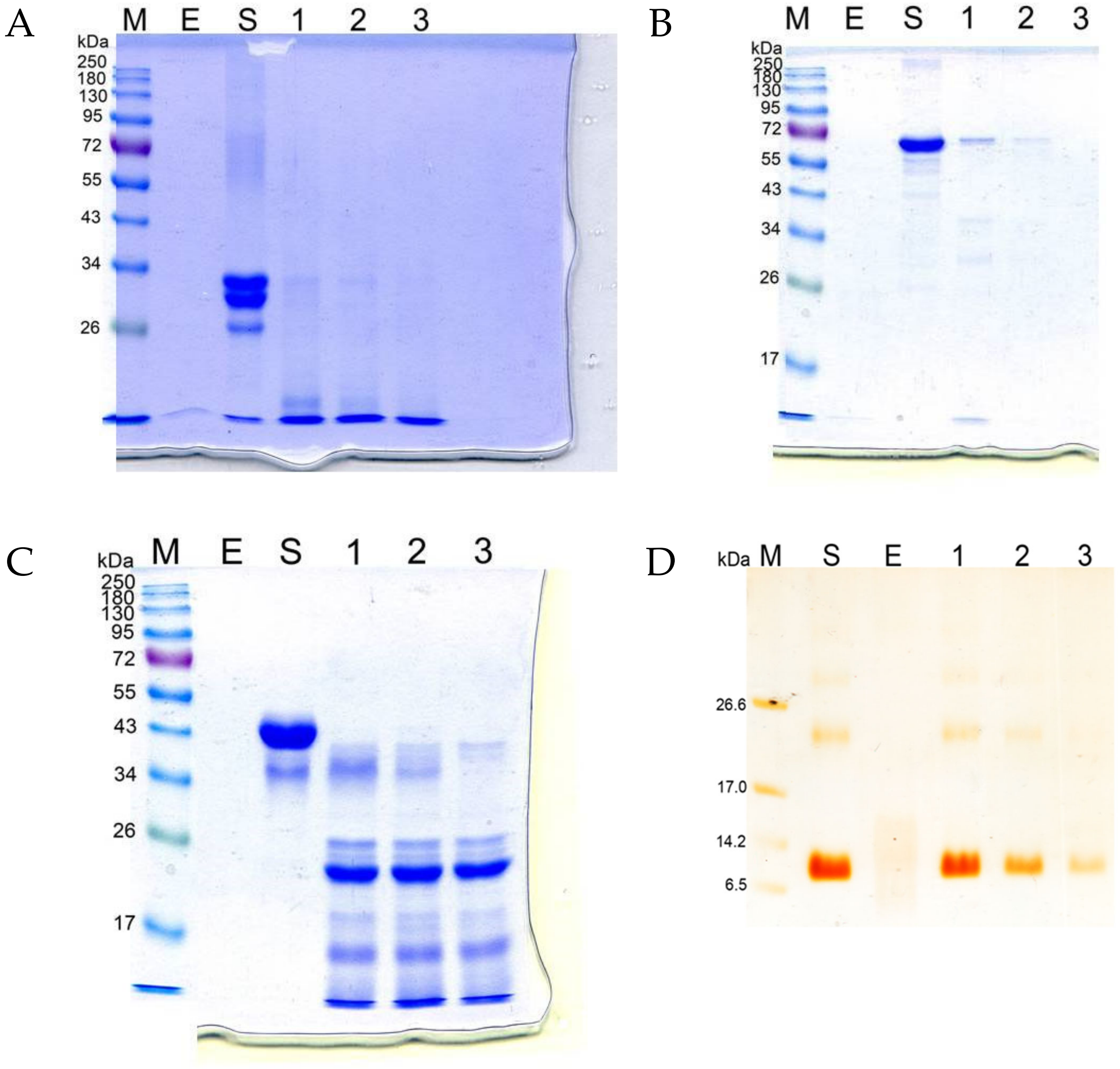

Figure S2. Degradation of the casein sodium salt (**A**), BSA (**B**), ovalbumin (**C**), and β-keratin (**D**) by the enzymatic extract from *Bacillus* sp. A5.3 depending on incubation time. M, protein molecular weight markers. E, enzymatic extract without a substrate; S, substrate. In (**A**): lane 1, 15s; lane 2, 30s; and lane 3, 60s. In (**B**): lane 1, 5 min; lane 2, 15 min; and lane 3, 60 min. In (**C**): lane 1, 1 min; lane 2, 5 min; and lane 3, 15 min. In (**D**): lane 1, 30 min; lane 2, 60 min; and lane 3, 120 min.
